# Reinitiation at non-canonical start codons leads to leak expression when incorporating unnatural amino acids

**DOI:** 10.1038/srep11866

**Published:** 2015-07-08

**Authors:** Tanja Kalstrup, Rikard Blunck

**Affiliations:** 1 Groupe d’Études des Protéines Membranaires (GÉPROM), Departments of Physics and of Physiology, Université de Montréal, Montréal, QC, Canada

## Abstract

With the rapid development of a continuously growing selection of unnatural amino acids (UAAs), UAA insertion becomes increasingly popular for investigating proteins. However, it can prove problematic to ensure the homogeneity of the expressed proteins, when homogeneity is compromised by “leak expression”. Here, we show that leak expression may be mediated by reinitiation and can result in unwanted proteins when stop codons for UAA insertion are mutated into the N-terminus of proteins. We demonstrate that up to 25% of leak expression occurs through reinitiation in the Shaker-Kv channel when stop codons are located within the first 70 amino acids. Several non-canonical start codons were identified as translation reinitaition sites, and by removing the start codons, we were able to decrease leak expression to less than 1%. Our study emphasizes the need to carefully inspect for leak expression when inserting UAAs and demonstrates how leak expression can be eliminated.

Genetic incorporation of unnatural amino acids (UAA) is a powerful tool to investigate protein structure and function. Through nonsense suppression of stop codons, UAAs are incorporated into proteins using chemically-charged tRNAs or orthogonal tRNA/tRNA synthetase (tRNA/RS) pairs^1^. With this method, numerous UAAs with different chemical and biophysical properties have been used as probes in *E. coli*, yeast, plant, amphibians and mammalian cells^1^,^2^. When using UAAs, it is crucial to have control of the protein translation process in order to get reliable results. Indeed, the orthogonal tRNA/RS pair provides site-specific incorporation of the UAA, with no or only a negligible fraction of unwanted proteins lacking the UAA, which may result from crosstalk with endogenous tRNAs or synthetases.

Also cellular mechanisms can generate leak expression when using UAA mutagenesis, in spite of the orthogonality of the tRNA/RS pair. Eukaryotic cellular mRNA is mainly translated via a linear scanning model which consists of initiation, elongation and termination. During initiation, the 40S ribosomal subunit is equipped with initiation factors and Met-tRNA, and binds to the 5’-terminal mRNA 7-methylguanosine cap structure (Fig. 1A). The complex then scans in the 5’ to 3’ direction in a base-by-base inspection until it encounters the first ATG start codon, where elongation begins with recruitment of the large 60S subunit and release of initiation factors^3^ (Fig. 1A). When the ribosome encounters a stop codon during elongation, translation is terminated. The ribosome disassembles from the mRNA and prepares for a second round of translation.

However, under some circumstances, translation does not strictly follow the linear scanning model. One example is stop codon *readthrough*, where a tRNA inserts its amino acid at the stop codon^4^, or the stop codon can be ignored due to ribosomal sliding^5^. The efficiency of stop codon readthrough includes complex combinations of stop codon identity, sequence context and RNA structure^6^,^7^,^8^, and such recoding strategies are especially used by viruses to generate a rich and varied protein synthesis. Therefore, translational stop codon readthrough is best understood in viruses, but has also been observed in yeast, *Drosophila* and humans^9^,^10^. Another recoding strategy is *reinitiation*, where multiple initiation sites can result in mRNAs harboring more than one open reading frame (ORF)^11^. If the eukaryotic ribosome encounters a stop codon shortly after initiation, a fraction of the initiation factors are still associated to the ribosome^12^,^13^. This allows the 40S subunit to remain on the mRNA and as a result, the 40S subunit continues scanning for a downstream start codon and reinitiates translation, resulting in a downstream ORF (dORF). Reinitiation only occurs when the distance between the first start codon and the next stop codon is short^13^,^14^, likely increasing the probability that initiation factors are still present. As a result, N-terminal truncated proteins can be translated from the upstream ORF (uORF). In eukaryotes, the ribosomal initiation complex involves 13 initiation factors (eIFs)^15^ but the identity and number of eIFs required for reinitiation is unknown. Reinitiation occurs in viruses^16^ and throughout the eukaryotic kingdom^17^,^18^,^19^. Reinitiation also occurs in prokaryotes, but the mechanism is different due to a distinct initiation machinery^17^.

In contrast to readthrough, reinitiation is more common in eukaryotes, and approximately 40% of mammalian mRNAs have a short uORF, although not all of them necessarily involve translation of both ORFs^20^,^21^. Examples of functional reinitiation at downstream methionines include regulation of Aquaporin-4 isoforms^22^ and LQT2 mutations in hERG channels leading to altered gating properties^23^ and defective trafficking^24^.

Previously, we successfully incorporated a fluorescent UAA into the Shaker voltage-gated potassium (Kv) channel^2^. The nonsense stop codons were located either central or C-terminal, and no leak expression was detected in the absence of the UAA, reflecting a strong orthogonality of the tRNA/RS pair used. Here, we show that when stop codons are introduced in the region near the N-terminus of the same Kv channel, a considerable amount of leak expression results from translation reinitiation which do not reinitiate at downstream methionines, but rather uses non-canonical (non-AUG) start codons.

The Kv channel is a homotetramer where each subunit consists of six transmembrane segments (S1–S6) containing the voltage sensor (S1–S4) and the pore (S5–S6). The N-terminal region (amino acids 1–46) of each subunit is a cytosolic soluble “ball-and-chain” region responsible for fast N-type inactivation (Fig. 1B)^25^,^26^, where the N-terminus move into the pore region and occludes the pore in response to sustained depolarization (Fig. 1C). When the N-terminus of a Shaker Kv channel is deleted (∆6-46), the inactivation is disrupted such that the channel remains open (Fig. 1C)^25^. The different current phenotypes of full length and N-terminal deletion channels allowed us to determine the absence or presence of the N-terminus in various Shaker mutants expressed in Xenopus oocytes using the cut-open voltage clamp technique^27^.

## Results

### Functional channels are translated despite stop codons in the N-terminus

**Figure 1:**
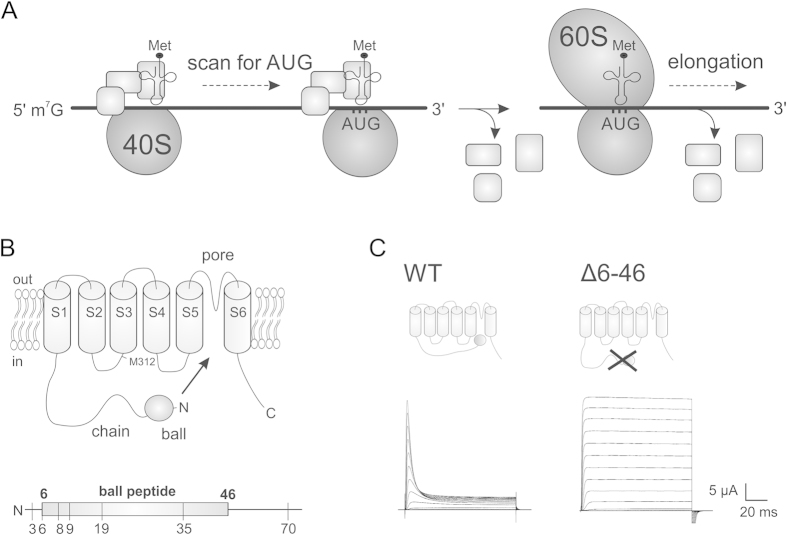
**A**) Simplified model for translation initiation in eukaryotes. First, the 40S small ribosomal subunit associates with initiation factors (shown as squares) and Met-tRNA^Met^ on the mRNA 5’ cap structure. The assembled complex then scans the mRNA sequence until it meets the first AUG codon, after which a fraction of initiation factors are released and the 60S large ribosomal subunit is recruited. Elongation of peptide bonds can then begin and the rest of the initiation factors are released. For a more detailed view see Jackson, *et al.*^15^. **B**) Topology of the Shaker Kv channel (top) and the N-terminal (below) with annotated positions used in this study. **C**) Ionic currents from WT and ∆6-46 Shaker channels upon depolarization from −100 mV to +60 mV in steps of +10 mV from a holding potential of –90 mV. The corresponding structures of each constructs are shown.

**Figure 2:**
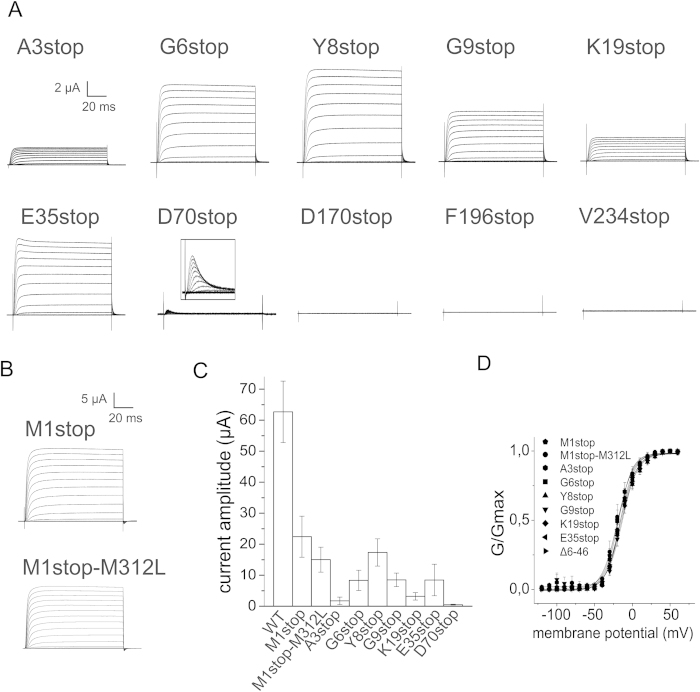
**A–B**) Ionic currents from Shaker mutants obtained with the same protocol as in 1C. **C**) Comparison of peak current amplitudes at +60 mV. Error bars indicate SEM with n =10–20 oocytes. **D**) Conductance-voltage (GV) relationships were fitted to a Boltzmann equation (V_½,M1stop_ = −19.1 mV, dV_M1stop_ = 9.9 mV, V_½,M1stop-M312L_ = −23.0 mV, dV_M1stop-M312L_ = 11.3 mV, V_½,A3stop_ = −18.1 mV, dV_A3stop_ = 9.1 mV, V_½,G6stop_ = −15.5 mV, dV_G6stop_ = 9.7 mV, V_½,Y81stop_ = −14.6 mV, dV_Y8stop_ = 10.8 mV, V_½,G9stop_ = −13.0 mV, dV_G9stop_ = 10.0 mV, V_½,K19stop_ = −16.3 mV, dV_K19stop_ = 9.3 mV, V_½,E35stop_ = −14.5 mV, dV_E35stop_ = 10.1 mV, V_½,∆6-46_ = −15.8 mV, dV_∆6-46_ = 10.6 mV).

In contrast to our previous findings where we introduced stop codons in the central and C-terminal part of the protein^2^, leak expression was present when stop codons were inserted within the first 70 amino acids in the Shaker Kv channel (A3stop, G6stop, Y8stop, G9stop, K19stop, E35stop and D70stop, Fig. 1B, Fig. 2A). No leak expression was measured for any stop codons tested downstream of residue D70 (L170stop, F196stop and V234stop shown in Fig. 2A, and A359stop and H486stop in Kalstrup & Blunck 2013^2^), which indicates that the mechanism responsible for leak expression is specific for N-terminal positions. D70stop expressed less than 1% whereas the other mutants expressed between 5 and 25% (Fig. 2C).

The leak expression presented in Fig. 2A occurs in the presence of the channel RNA only, and the mechanism leading to expression here therefore cannot be attributed to crosstalk between the modified tRNA/synthetase pair and endogenous amino acids. The observed leak expression could be due to readthrough or translational reinitiation beyond the inserted stop codon. One indication was the dependence of leak expression on the position of the stop codon. When stop codons are inserted proximal to the N-terminus, a short uORF is generated in the case of reinitiation. Due to the short length of the uORF, the likelihood is high that (some of) the initiation factors are still attached, allowing a second initiation to take place at the next start codon. The further downstream a stop codon is located, the longer the translated uORF will be and the higher will be the chance that all initiation factors separated from the ribosome, such that reinitiation cannot occur. Readthrough, on the other hand, is not dependent on the presence of initiation factors and should have no dependence on the relative position. We only found leak expression for N-terminal stop codons, pointing towards reinitiation as the prevalent mechanism for leak expression.

We were able to further corroborate this indication by investigating expression of the uORF. In the Shaker Kv channel, used in this study, we were also able to determine whether the uORF has been expressed because the N-terminus forms the ball peptide responsible for N-type inactivation and its presence is visible in the functional data. Deletion of residues 6–46 removes inactivation (Fig. 1C) while synthetic N-terminal Shaker peptides of the first 20 amino acids can restore inactivation in ∆6-46 channels^28^.

In the case of reinitiation, an N-terminal peptide and an N-terminal truncated channel are translated. Depending on the length of the peptide and where the second translation begins, such N-terminal truncation could lead to disrupted N-type inactivation. E35stop and D70stop exhibited weak and complete N-type inactivation, respectively, while the other mutants did not undergo any N-type inactivation and were identical to ∆6-46 channels (Figs 1C and 2A,D). It seems that the uORFs generated in E35stop and D70stop were responsible for the inactivation of truncated channels, while the uORFs in the other mutants were too short to inactivate (<19 amino acids, Fig. 1B). We elaborate on this in the next section.

Principally, inactivation in E35stop and D70stop could also be due to stop codon readthrough, where full length channels are translated, and thus undergo N-type inactivation. However, readthrough would also have led the mutants with a shorter uORF to inactivate (Fig. 2A). In addition, removal of the start codon (M1stop) did not reduce leak expression (Fig. 2B–C), indicating that ribosomes are indeed capable of initiating translation downstream in the sequence. Taken together, these results indicate that the most likely mechanism causing leak expression here is reinitiation. The question remained at what site translation was reinitiated.

One possibility would be initiation at a downstream methionine; it was for instance previously reported that Kv1.4 channels with a stop codon at residue 19 exhibited leak currents from truncated channels which were eliminated by removal of a methionine at residue 108^29^. However, here, removal of the next downstream methionine (M312L) in Shaker did not significantly influence leak expression (Fig. 2B). We ignored later methionines since the resulting translated protein would not yield functional channels, lacking most of or the entire voltage sensor. Reinitiation thus has to occur at sites other than the standard start codon AUG.

### Non-canonical start codons are used as translation reinitiation sites

**Figure 3:**
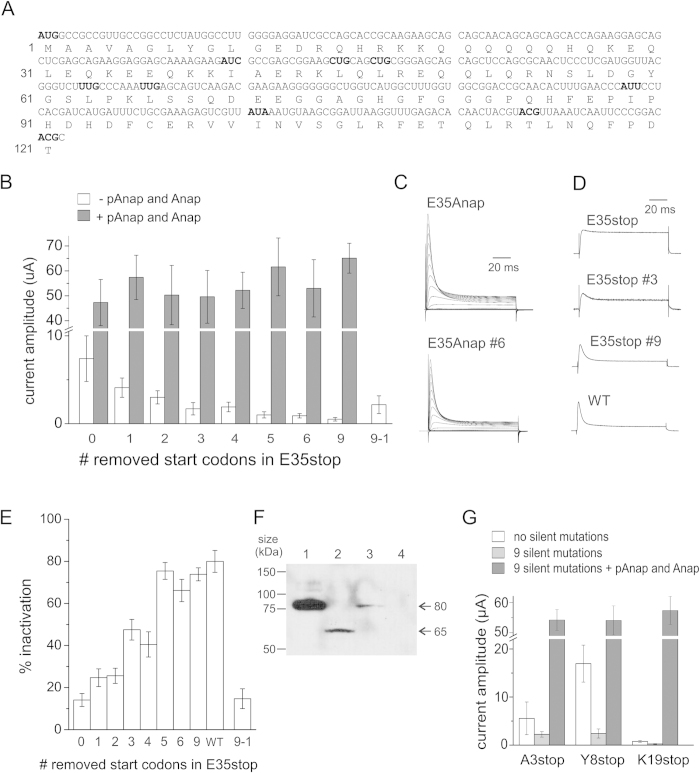
**A**) Sequence of amino acids 1−121 and the corresponding mRNA nucleotides in Shaker. The nucleotides of the canonical start codon (M1) and the following 9 downstream non-canonical start codons (I40, L45, L47, I89, L36, L66, I101, T114, and T121) are shown in bold. AAG and AGG codons were ignored. Numbers refer to the amino acid sequence. **B**) Comparison of peak current amplitudes at +60 mV of E35stop with silent mutations, without and with Anap incorporation. Numbers refer to the number of mutated start codons such that 1 is E35stop-L45L, 2 is E35stop-L45L-L47L, 3 is E35stop-I40L-L45L-L47L, 4 is E35stop-I40L-L45L-L47L-L63L, 5 is E35stop-I40L-L45L-L47L-L63L-L66L, 6 is E35stop-I40L-L45L-L47L-L63L-L66L-I89L and 9 is E35stop-I40L-L45L-L47L-L63L-L66L-I89L-I101L-T114T-T121T. Error bars indicate SEM with n = 20–30 oocytes. **C**) Inactivation is recovered with Anap incorporation. Shown are examples of ionic currents from E35Anap and E35Anap #6 (six mutated start codons). Voltage protocols are the same as in Fig. 1. **D**) Comparison of inactivation measured as the ratio between the current amplitude at the end of the +60 mV pulse, and the maximum current amplitude. Error bars indicate SEM with n = 10–20 oocytes. **E**) Depolarization from −90 mV to +60 mV is shown for E35stop mutants and WT showing how inactivation increases when channel expression decreases. **F**) Western blot of isolated Xenopus oocytes membranes expressing Shaker WT (lane 1, 30 oocytes), E35stop (lane 2, 70 oocytes), E35Anap (lane 3, 30 oocytes) and non injected (lane 4, 70 oocytes). **G**) Comparison of peak current amplitudes at +60 mV for A3stop, Y8stop and K19stop, and removal of 9 start codons without and with Anap incorporation. Error bars indicate SEM with n = 5–10 oocytes.

Increasing evidence from the literature has underscored the use of non-canonical start codons which deviate from the canonical AUG codon by one nucleotide, except AAG and AGG^30^,^31^,^32^. We therefore screened the Shaker mRNA sequence for such non-canonical codons and found 9 in-frame codons within the first 121 amino acids at position I40, L45, L47, L63, L66, I89, I101, T114 and T121 (highlighted in bold in Fig. 3A). To verify that these codons were used as start codons in reinitiation, silent mutations were introduced into the E35stop background. First, we mutated L47 (#1 in Fig. 3A), then both L47 and L45 (#2) and then L47, L45 and I40 (#3). Each mutation led to a decreased expression level in a stepwise manner (Fig. 3B). Therefore, all ribosomes do not nessecarily reinitiate at the first codon they encounter (I40). For the subsequent mutations of L63 (#4), L66 (#5) and I89 (#6), only #5 led to a decreased expression. When the last three codons were removed in addition (#9), expression levels further decreased. Expression could partially be restored upon reinsertion of I40 into the #9 background (#9-1) confirming that reinitiation is not limited to the first start codon. As a control, the unnatural amino acid Anap was incorporated in response to the stop codon using the orthogonal tRNA/Anap-RS pair (pAnap)^2^,^33^,^34^. With Anap, wildtype expression levels and N-type inactivation were fully recovered, reflecting full length channels (Fig. 3B–C). The expression levels with Anap did not decrease with the removal of start codons, excluding the possibility that RNA stability might play a role in the decreased expression. If the RNA had become instable by silently removing the non-canonical start codons, also expression in presence of Anap would have been reduced. Therefore, our experiments demonstrate that several non-canonical start codons in the region of amino acids 40–121 are recognized by the ribosome to reinitiate translation in E35stop channels.

The sequence context of start codons has significant influence on the initiation efficiency of both canonical and non-canonical start codons^35^,^36^. The Kozak consensus sequence gccRccAUGG is the optimal context in eukaryotes, with the nucleotides in capital letters at position −3 and +4 (relative to AUG) being most important. The purine (R = A or G) at −3 is more important than a G at +4, and the other nucleotides only play a role if there is neither a purine at −3 or a G at +4^37^. For the 9 codons tested here, L47, L63 and I89 have the weakest Kozak sequence (Fig. 3A), which could explain why L63 and I89 are not used as initiation sites. The codon for L47 (CTG) is one of the most probable non-AUG codons^30^ and may explain why it is recognized by the ribosome despite a weak Kozak sequence.

When we reduce the translation efficiency of reinitiated channels, only the dORF is affected, while the short uORF is not. Therefore, the ratio of free N-terminal peptides to truncated channels will become larger when expression of the truncated channels decreases. This would explain why the slight inactivation of E35stop channels increases and almost reaches WT levels when the alternative start codons are removed (Fig. 3D–E). These findings are supported by the fact that the efficiency of N-type inactivation indeed depends on both the length and the concentration of the N-terminal peptide^28^. We estimated the intracellular concentration of the uORF (amino acids 1–34) to be at least 2 μM (see Methods), which is in agreement with previous peptide concentrations, where application of free peptides (amino acids 1–20) to ∆6-46 Shaker channels resulted in partial and full inactivation at concentrations of 10 μM and 100 μM, respectively^28^. Alternatively, the residual expression could be caused by readthrough, which would also show complete inactivation.

To confirm protein truncation in E35stop, a Western blot was performed for direct visualization of protein lengths with C-terminal myc-tagged Shaker constructs (Fig. 3F). Full-length wildtype Shaker exists in core-glycosylated (~80 kDa) and mature (~110 kD) form^38^,^39^. For wildtype and E35Anap, we mainly see the core-glycosylated band at 80 kDa since the mature form only appears after longer expression times. E35stop in contrast, shows one band at ∼65 kDa. At 65 kD, we can exclude the possibility that E35stop leads to inefficient glycosylation as unglycosylated Shaker channels migrate at ∼75 kDa^38^.

The expected truncated proteins can be ordered into two groups: Those which reinitiate at I40, L45, L47 or L66 would theoretically be ∼5–7 kDa shorter than wildtype. Those which reinitiate at I101, T114 or T121 would be ∼11–14 kDa shorter than wildtype. We only observed a single band at 65 kDa, despite loading 10 times more of E35stop on the SDS-PAGE gel compared to wildtype (not shown). The band at 65 kDa agrees with core-glycosylated truncated channels, reinitiating at I101, T114 and T121. From reinserting only the first non-canonical start codon, we know, however, that also the first start codons are used. It is possible that low expression combined with different constructs resulted in protein concentrations too low to detect on a western blot. Alternatively, the small differences in length may result in truncated versions to migrate collectively.

To verify that reinitiation resulted in leak expression in the other mutants, we removed the same 9 alternative start codons in A3stop, Y8stop and K19stop. The expression levels decreased for all three mutants when removing the alternative start codons in the same manner as for E35stop (Fig. 3F). In addition, Anap incorporation rescued succesfully the full length channels with WT expression levels in presence of the 9 silent mutations, confirming that reinitiation is a general mechanism causing leak expression.

## Discussion

In this study, we have identified translation events which require attention when UAAs are incorporated specifically in the N-terminal region of a protein. The efficient Xenopus oocyte expression system and the high sensitivity of electrophysiological recordings allowed us to measure low expression levels (<5%) which typically are difficult to resolve without direct access to protein function. Our system also allowed to efficiently discriminate between truncated and full-length proteins.

Based on the expression levels in Fig. 2C, we estimate that reinitiated translations can generate 5–25% leak expression when the length of the uORF is between 3 and 35 codons. Since *cis*-acting mRNA sequences and RNA structure can affect reinitiation efficiency^13^,^40^, the lengths mentioned here cannot be exactly extrapolated to other proteins and remain approximations. Our results are consistent with studies of the length and time dependence of reinitiation at downstream methionines, where expression levels decreased to 30% in the presence of a 13-codon uORF^13^, or to 50% in the presence of a 28-codon uORF^14^. The same studies showed that reinitiation was optimal for a certain uORF length, and reduced gradually when the uORF was lengthened further, most likely due to loss of initiation factors. Examples include reduction in reinitiation when a 13-codon uORF was lengthened to 33 codons^13^, or a complete inhibition when a 24-codon uORF was lengthened to 40 codons^14^. These reported magnitudes of reinitiation inhibition by uORF lengths also correlate with our results, where reinitiation decreased to less than 1% when the uORF was lengthened to 70 codons (Fig. 2C). Our results thus indicate that the nature of the reinitiation site (canonical versus non-canonical) does not play a major role.

Leak expression was reduced from 14% to less than 1% in E35stop channels when 9 non-canonical start codons between codon 40 and 121 were removed. Although the remaining leak expression was very subtle, it means nevertheless that the 40S ribosomal subunit is capable of scanning at least 258 nt (the length in nucleotides between residue 35 and 121) for a downstream non-canonical start codon. Comparison with scanning lengths for reinitiation at methionines shows similar results with 63 nt and 129 nt in hERG^23^,^24^ and 270 nt in Kv1.4 channels^29^.

Kozak showed that reinitiation does not happen when the distance between the stop codon and the next start codon is short (ie. 8 nt) and that reinitiation is not limited to the closest downstream ATG codon^11^, because the 40S subunit only gradually becomes capable of reinitiation. This is confirmed by our results for non-canonical start codons, which show that the first start codon (I40) is only used by a fraction of the ribosomes, while others continue scanning to reinitiate at later start codons.

Our results let us conclude that the established conditions for reinitiation at methionines also apply to non-canonical start codons. Taken together, the likeliness for reinitiation is balanced between the length of the uORF (optimal length is 2–50 codons), the scanning length between the stop and the next start codon (between 8 and 300 nt) and the Kozak sequence context. During the first initiation process, the 40S subunit scans base-by-base for a start codon. It is therefore likely that during scanning for reinitiation, the 40S subunit also scans base-by-base and thus has no memory of the codon-frame of the uORF. As a result, it is not unlikely that out-of-frame start codons can serve as reinitiation sites. In that case, the translated dORF would be non-functional and thus be of no concern for UAA experiments. In the quadruplet codon technique for UAA insertion^41^, a frame-shift occurs during termination, but since the 40S subunit does not scan in codons, but in a base-by-base fashion, reinitiation is likely equally prone to occur.

In cases where full length and N-terminal truncated proteins are functionally identical, it can be difficult to differentiate between stop codon readthrough and reinitiation when leak expression exists in the absence of both UAA and the tRNA/RS pair. With the present study, we emphasize the importance of translation reinitiation when using UAA mutagenesis. Since reinitiation only occurs when the uORF is short, the issue is particularly relevant when UAAs are incorporated near the N-terminus of the protein of interest. Furthermore, we underline that attention should not be limited to downstream methionines, but that non-canonical start codons also serve as reinitiation sites.

Our results provide evidence that when modifying the genetic code for incorporation of UAAs, other cellular mechanisms may be affected as well. The high level of regulation at the translation initiation step is sensitive to minimal changes at the 5’ end of the mRNA sequence in terms of codons, sequence context and secondary structure. As a result, all these factors must be considered when inserting UAAs into the first 100 amino acids of any protein and especially in proteins where the function is not as readily visible as in ion channels.

It is possible that the efficiency of reinitiation-mediated leak expression is lower in the presence of UAAs than in their absence, and as a consequence low enough to be neglected. Nevertheless, in order to avoid N-terminal truncated proteins and proteins lacking the UAA to co-express with the UAA containing protein – in particular in multimeric proteins, – caution should be exercised against reinitiation, and removal of downstream start codons may decrease or eliminate such leak expression.

## Methods

### Molecular Biology and Oocyte Injection

All mutations were introduced into the *Drosophila* Shaker H4 gene in the pBSTA vector^42^. The tRNA/AnapRS synthetase pair were encoded on a cDNA (pAnap) with CMV promoter for the synthetase^2^; kind gift of P.G. Schultz, Scripps Research Institute. *Xenopus laevis* oocytes were injected with 35ng of *in vitro* transcribed RNA and incubated for 2 days at 18 °C in Barth solution. For incorporation with Anap, 9.2 nl of 0.1 ng/nL cDNA encoding the tRNA/AnapRS pair^2^ was injected into the oocyte nucleus 6–24 hours prior to coinjection of 23 nL 1 mM Anap and 35 ng RNA. For leucine and threonine silencing, CTC and ACT codons were used, respectively. Isoleucines were mutated to leucines using CTC, since all isoleucine codons are potential start codons. The codon used for the M312L mutation was CTC.

### Electrophysiology

Voltage clamp was performed with a CA-1B amplifier (Dagan). Currents were recorded in the cut-open oocyte voltage-clamp configuration as described^43^ and analyzed by using GPatch (Department of Anesthesiology, University of California, Los Angeles). Linear capacitance currents were subtracted online using the P/4 protocol^44^. The external solution contained in mM: 5 KOH, 110 NMDG, 10 Hepes, and 2 Ca(OH)_2_, and the internal solution contained in mM: 115 KOH, 10 Hepes, and 2 EDTA. Both solutions were adjusted to pH 7.1 with MES. Conductance (G) was calculated from the steady state currents (I) via G = I/(V−V_rev_), where V_rev_ is the reversal potential. Conductance-voltage relationships (GV) were fitted to a Boltzmann relation of the form G/G_max_ = (1 + exp(−(V −V_1/2_)/dV))^−1^.

### Estimation of the intracellular inactivation peptide concentration

To estimate a lower limit for the concentration of the intracellular inactivation peptides, we determined how many of the original constructs (Shaker-W434F) express under identical conditions. The N-terminal peptide should, as a short cytosolic peptide, reach at least the same concentration. The number of intracellular uORFs (the number of N-termini) per channel equals the number of subunits. The number of subunits *n*, expressed at the surface, can be calculated using 3.3 elementary charges per subunit^45^,^46^,^47^ 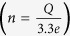, where Q is the total gating charge (26 nC) obtained with cut-open voltage clamp after 2 days of RNA incubation with Shaker-W434F. Since only part of the oocyte is clamped in cut-open voltage clamp, the ratio of whole oocyte surface to clamped sherical cap surface was calculated using an oocyte radius of 0.5 mm and an angle of 30° from the center of the oocyte to the spherical cap edge. The final concentration of N-termini is then given by equation 1, where *V* is the oocyte volume (0.52 μl) as calculated from the radius.


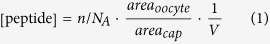


### Western blot

For detection of Shaker, we generated myc-tagged Shaker constructs with Glu-Gln-Lys-Leu-Ile-Ser-Glu-Glu-Asp-Leu inserted in the COOH terminus (after Val650). Oocytes were injected with myc-Shaker RNA and incubated at 18 °C for 4 days. Membrane isolations were optained as previously described with minor modifications^48^ 30–70 oocytes were homogenized in 1 ml HEDP buffer (100 mM HEPES, 1 mM EDTA, pH 7.6 with NaOH) supplemented with 0.5 mM phenylmethanosulfonyl fluoride, 0.5 ug/ml aprotonin, 0.5 ug/ml leupeptin and 0.7 ug/ml pepstatin, and centrifuged at 3.000 *g* for 10 min. All steps were performed at 4°C. The lipid layer on the surface was removed, and the supernatant centrifuged at 18.000 g for 30 min. The pellet was suspended in external recording solution and 4xSDS sample buffer with 50 mM DTT, and incubated for 10 min at 70°C. The samples were loaded on an 8% SDS gel. Running buffer contained 190 mM glycine, 25 mM Tris base and 0.1% SDS for electrophoresis. Precision Plus Protein Standard (Bio-Rad) was used as protein weigth marker. Wet transfer onto a nitrocellulose membrane was performed with a transfer buffer containing 190 mM glycine, 25 mM Tris base and 20% methanol. The membrane was then incubated with the anti-myc antibody (Invitrogen) and protein was detected using SuperSignal West Dura Substrate (Invitrogen).

## Additional Information

**How to cite this article**: Kalstrup, T. and Blunck, R. Reinitiation at non-canonical start codons leads to leak expression when incorporating unnatural amino acids. *Sci. Rep.* **5**, 11866; doi: 10.1038/srep11866 (2015).
